# Draft genome sequence of an arsenotrophic *Achromobacter aegrifaciens* strain isolated from soil in Bangladesh

**DOI:** 10.1128/mra.00137-24

**Published:** 2024-09-24

**Authors:** M. Nazmul Hoque, Anamica Hossain, Golam Mahbub Faisal, Momtaz Zamila Bukharid, M. Anwar Hossain, Munawar Sultana

**Affiliations:** 1 Department of Microbiology, University of Dhaka, Dhaka, Bangladesh; 2 Molecular Biology and Bioinformatics Laboratory, Department of Gynaecology, Obstetrics and Reproductive Health, Bangabandhu Sheikh Mujibur Rahman Agricultural University, Gazipur, Bangladesh; 3 Research Fellow, One Health Laboratory, icddr,b, Dhaka, Bangladesh; 4 Jashore University of Science and Technology, Jashore, Bangladesh; SUNY College of Environmental Science and Forestry, Syracuse, New York, USA

**Keywords:** draft genome, arsenotrophic, *A. aegrifaciens*, arsenic (As), pollution, soil

## Abstract

We report the draft genome of an arsenotrophic *Achromobacter aegrifaciens* BAS32 isolated from arsenic (As)-contaminated soil in Bangladesh. This genome contains several predicted gene clusters for As-conversion, namely, As resistance (*ars*HCsO), arsenite-oxidizing (*aio*BA), and arsenate-reducing (*ars*RCDAB) gene clusters along with antibiotic resistance genes (ARGs).

## ANNOUNCEMENT

*Achromobacter aegrifaciens* is widely distributed in various environments, including freshwater, groundwater, marine habitats, and soil (1, 2). Despite research on sulfur oxidation (3), its role in As detoxification remains unexplored. It is, however, tolerant to arsenite (As(III)), similar to arsenotrophs like *A. xylosoxidans* BHW-15 (4). Therefore, the genome *A. aegrifaciens* BAS32 was sequenced to elucidate its genomic potential for As detoxification.

*A. aegrifaciens* BAS32 was isolated from As-contaminated soil samples, collected from the Bogura (24.85° N, 89.37° E) district of Bangladesh. In brief, the soil sample (2 gm) was dissolved in 20 mL 0.9% NaCl, and 2 mL of resulting suspensions was incubated in 60 mL of liquid minimal salts medium (MSM) (5) for enrichment at 30°C for 7 days. Afterward, the enrichment broth was subjected to threefold serial dilution, plated onto heterotrophic MSM agar plates containing 2 mM of NaAsO2 for incubation at 25°C with a rotary shaker set at 120 rpm for 2 weeks (6), and pure colonies were screened following previously published protocols (6). Genomic DNA from the BAS 32 isolate was extracted from the pure colony by the FavorPrepTM Tissue Genomic DNA Extraction Mini Kit (FavorGen-Europe, Vienna), and purity and concentration of the extracted DNA were checked by NanoDrop 2000 UV–Vis Spectrophotometer (Thermo Fisher, USA). Libraries were generated from 1 ng of DNA using the Nextera DNA Flex Library Prep Kit (Illumina, USA), and whole-genome sequencing (WGS) was conducted using the Illumina NovaSeq PE150 sequencer (Illumina, San Diego, CA, USA) with a 2 × 250-bp protocol (7, 8). The paired-end raw reads (*n* = 16,443,452 bp) were trimmed using Trimmomatic v0.39 (9) to remove Illumina adapters, known Illumina artifacts and phiX, and quality-checked using FastQC v0.11.7 (10). Reads (*n* = 16,442,869) with phred scores > 20 were assembled using SPAdes v.3.12 (11), and quality of the assembled genome was checked using QUAST v.5.0.2 (12). Genome completeness was assessed through CheckM v1.2.2 using the *Achromobacter* CheckM marker set (13). The NCBI Prokaryotic Genome Annotation Pipeline v6.4 (14) was used for genome annotation. SimpleSynteny v1.6b (15, 16, 16) and Proksee (17) databases were used to predict As-transforming gene clusters, ARGs, and mobile genetic elements (MGEs) in the draft genome. We used default parameters for all software unless stated otherwise.

**Fig 1:**
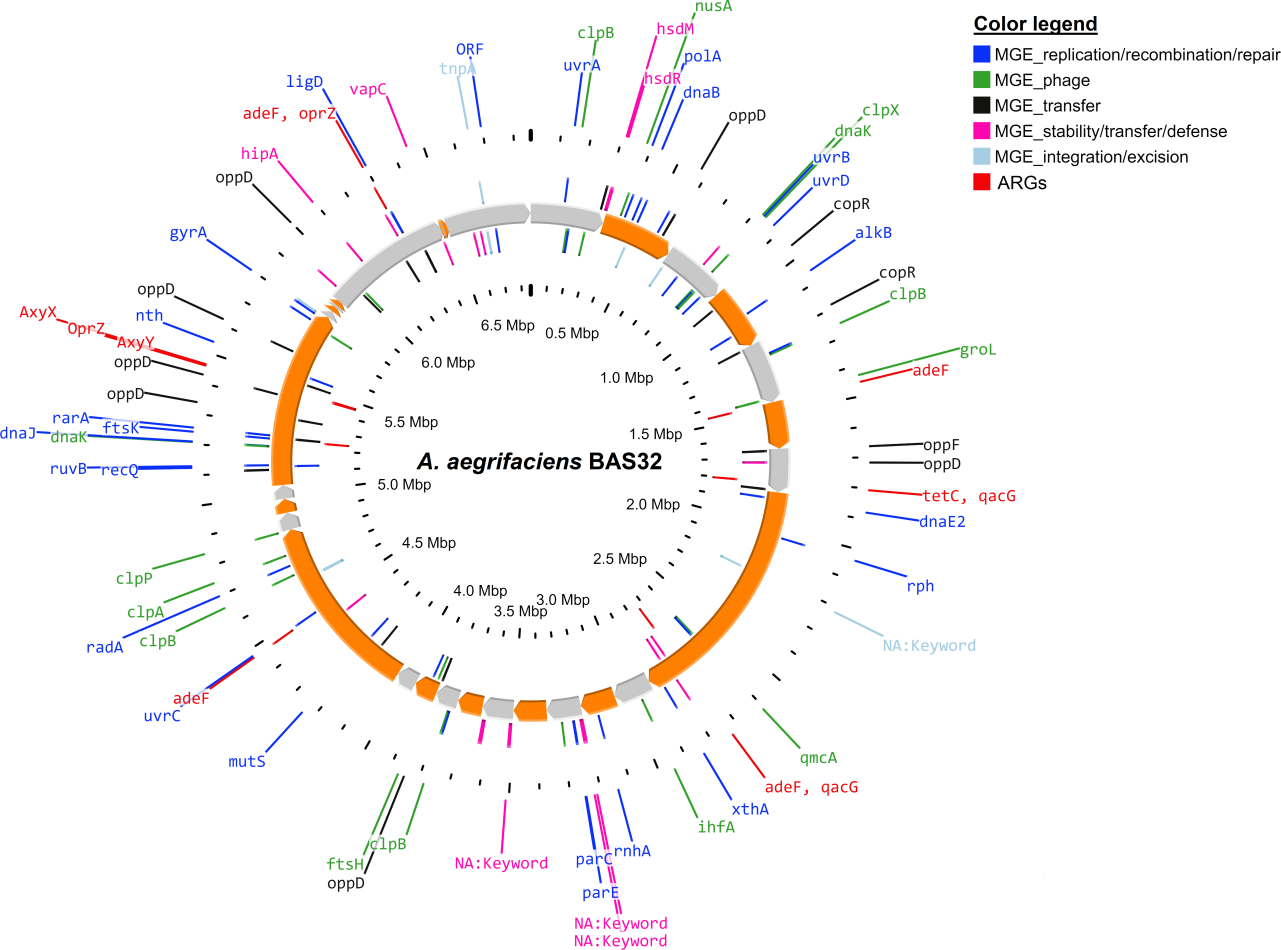
Circular genome representation of *A. aegrifaciens* BAS32 showing different antibiotic resistance genes (ARGs) and mobile genetic elements (MGEs). The orange and gray arrows in the middle represent the open reading frames (ORFs) of the genome. The circular genomic map was created using the Proksee tool available on the CGView server (https://cgview.ca/).

The draft genome of BAS32 comprised 33 contigs, with 43 x coverage and 98.11% completeness. The annotated genome length, GC content, and *N_50_* value were 6,857,615 bp, 65.5%, and 321,383 bp, respectively. The BAS32 genome contained 361 subsystems, 6,287 protein-coding genes, and 63 RNA genes (four rRNA, four ncRNA, and 55 tRNA). Three putative As-transforming gene clusters such as As(III) oxidizing (*aio*BA), arsenate reducing (*ars*RCDAB), and the MMA(III) oxidizing *ars* resistance gene (*ars*HCsO) clusters were identified in the BAS32 genome. The genome simultaneously harbored six ARGs (*ade*F, *tet*C, *qac*G, *axy*X, *axy*Y, and *opr*Z) and several mobile genetic elements (MGEs) (Fig. 1).

## Data Availability

The whole genome of the *Achromobacter aegrifaciens* BAS32 has been deposited at NCBI/GenBank under the accession number JAVCWD000000000, and the raw reads or SRA data can be cited under BioProject accession PRJNA1005075 and Sequence Read Archive (SRA) accession SRR26458878. The version described in this paper is version JAVCWD000000000.1.

## References

[1] Rahaman MS, Mise N, Ichihara S. 2022. Arsenic contamination in food chain in Bangladesh: a review on health hazards, socioeconomic impacts and implications. Hyg Environ Health Adv 2:100004. doi:10.1016/j.heha.2022.100004

[2] Brenner DJ, Krieg NR, Staley JT, Garrity G. 2005. Bergey’S manual of systematic bacteriology, volume 2: the proteobacteria, part C. Springer.

[3] Hutt LP, Harper GM, Moody AJ, Boden R. 2021. Insights into growth kinetics and roles of enzymes of Krebs’ cycle and sulfur oxidation during exochemolithoheterotrophic growth of Achromobacter aegrifaciens NCCB 38021 on succinate with thiosulfate as the auxiliary electron donor. Arch Microbiol 203:561–578. doi:10.1007/s00203-020-02028-1 32989476

[4] Diba F, Khan MZH, Uddin SZ, Istiaq A, Shuvo MSR, Ul Alam ASMR, Hossain MA, Sultana M. 2021. Bioaccumulation and detoxification of trivalent arsenic by Achromobacter xylosoxidans BHW-15 and electrochemical detection of its transformation efficiency. Sci Rep 11:21312. doi:10.1038/s41598-021-00745-1 34716390 PMC8556249

[5] Mishra S, Singh SN, Pande V. 2014. Bacteria induced degradation of fluoranthene in minimal salt medium mediated by catabolic enzymes in vitro condition. Bioresour Technol 164:299–308. doi:10.1016/j.biortech.2014.04.076 24862007

[6] Diba F, Hoque MN, Rahman MS, Haque F, Rahman KMJ, Moniruzzaman M, Khan M, Hossain MA, Sultana M. 2023. Metagenomic and culture-dependent approaches unveil active microbial community and novel functional genes involved in arsenic mobilization and detoxification in groundwater. BMC Microbiol 23:241. doi:10.1186/s12866-023-02980-0 37648982 PMC10466822

[7] Hoque MN, Jerin S, Faisal GM, Das ZC, Islam T, Rahman ANMA. 2023. Whole-genome sequence of multidrug-resistant Escherichia coli strains isolated from mice with mastitis. Microbiol Resour Announc 12:e0032023. doi:10.1128/mra.00320-23 37314348 PMC10353464

[8] Hoque MN, Moyna Z, Faisal GM, Das ZC, Islam T. 2023. Whole-genome sequence of multidrug-resistant Klebsiella pneumoniae MNH_G2C5, isolated from bovine clinical mastitis milk. Microbiol Resour Announc 12:e0007923. doi:10.1128/mra.00079-23 37093061 PMC10190633

[9] Bolger AM, Lohse M, Usadel B. 2014. Trimmomatic: a flexible trimmer for Illumina sequence data. Bioinformatics 30:2114–2120. doi:10.1093/bioinformatics/btu170 24695404 PMC4103590

[10] Andrews S. 2010. FastQC: a quality control tool for high throughput sequence data. Babraham Bioinformatics, Babraham Institute, Cambridge, United Kingdom.

[11] Bankevich A, Nurk S, Antipov D, Gurevich AA, Dvorkin M, Kulikov AS, Lesin VM, Nikolenko SI, Pham S, Prjibelski AD, Pyshkin AV, Sirotkin AV, Vyahhi N, Tesler G, Alekseyev MA, Pevzner PA. 2012. SPAdes: a new genome assembly algorithm and its applications to single-cell sequencing. J Comput Biol 19:455–477. doi:10.1089/cmb.2012.0021 22506599 PMC3342519

[12] Gurevich A, Saveliev V, Vyahhi N, Tesler G. 2013. QUAST: quality assessment tool for genome assemblies. Bioinformatics 29:1072–1075. doi:10.1093/bioinformatics/btt086 23422339 PMC3624806

[13] Parks DH, Imelfort M, Skennerton CT, Hugenholtz P, Tyson GW. 2015. CheckM: assessing the quality of microbial genomes recovered from isolates, single cells, and metagenomes. Genome Res 25:1043–1055. doi:10.1101/gr.186072.114 25977477 PMC4484387

[14] Tatusova T, DiCuccio M, Badretdin A, Chetvernin V, Nawrocki EP, Zaslavsky L, Lomsadze A, Pruitt KD, Borodovsky M, Ostell J. 2016. NCBI prokaryotic genome annotation pipeline. Nucleic Acids Res 44:6614–6624. doi:10.1093/nar/gkw569 27342282 PMC5001611

[15] Veltri D, Wight MM, Crouch JA. 2016. SimpleSynteny: a web-based tool for visualization of microsynteny across multiple species. Nucleic Acids Res 44:W41–W45. doi:10.1093/nar/gkw330 27141960 PMC4987899

[16] Alcock BP, Huynh W, Chalil R, Smith KW, Raphenya AR, Wlodarski MA, Edalatmand A, Petkau A, Syed SA, Tsang KK, et al.. 2023. CARD 2023: expanded curation, support for machine learning, and resistome prediction at the comprehensive antibiotic resistance database. Nucleic Acids Res 51:D690–D699. doi:10.1093/nar/gkac920 36263822 PMC9825576

[17] Grant JR, Enns E, Marinier E, Mandal A, Herman EK, Chen C-Y, Graham M, Van Domselaar G, Stothard P. 2023. Proksee: in-depth characterization and visualization of bacterial genomes. Nucleic Acids Res 51:W484–W492. doi:10.1093/nar/gkad326 37140037 PMC10320063

